# Supplementary material for: Hormone Regulation Effect of Blue Light on Soybean Stem Internode Growth Based on the Grey Correlation Analysis Model
Source: Int J Mol Sci. 2025 May 6;26(9):4411. doi: 10.3390/ijms26094411 (PMC12072184; doi:10.3390/ijms26094411)
Supplement: Supplementary file 1 [file ijms-26-04411-s001.zip › Supplementary material S1.pdf]

Quantitative PCR (qPCR) analysis:

Total RNA was isolated with Trizol (Thermo Fisher Scientific) according to the manufacturer's instructions. 100 ng^{-1} mg of RNA was reverse transcribed to cDNA with random RNA-specific primers using the highcapacity cDNA reverse transcription kit (Applied Biosystems, USA). An Eppendorf Master Cycle Realplex2 and a SYBR Green PCR Master Mix (Applied Biosystems) were used for real-time PCR (40 cycles).

Table S2 The primer sequences of target genes used for PCR

Target genes	Primer sequences
Actin-F	5'-CCATAAACGATGCCGACCAG-3'
Actin-R	5'-AGCCTTGCGACCATACTCCC-3'
phyA1-F2	5'-ATAGTTGCCACGCGCAGTAC-3'
phyA1-R2	5'-AAACCCCAAAGTCTCTCCCTC-3'
phyB-like-F2	5'-CGTTGGAGAAGCGGGTTT-3'
phyB-like-R2	5'-AGTCACACCCAACGGATAATAGT-3'
PIF4A-F1	5'-CAGGAGCAAGATGTAAGCAGG-3'
PIF4A-R1	5'-AATCGGTCTTTGGACATAATCTC-3'
PIF4B-F1	5'-TCCTGATTCATCCCAGAAAGAC-3'
PIF4B-R1	5'-CCCTGCTTACATCTTTCTCCTG-3'
GA20ox1-F1	5'-GAAATGAGTCGGTTATGAGGTTG-3'
GA20ox1-R1	5'-GTGGAGAATGGTTAGGGATGTAG-3'
GA2ox4-F1	5'-TCGGACCCACCCTTTCAC-3'
GA2ox4-R1	5'-CTTCCCTCACAACCTCATCG-3'
LOC100783826-F2 (HY5)	5'-TCAAGAGGTTGTTGCGGAATA-3'
LOC100783826-R2	5'-TGCTTTTGTTCAATGCTGTCA-3'
LOC100799695-F2 (DELLA)	5'-CATCGTGGAACAAGAAGCG-3'
LOC100799695-R2	5'-GACATTAACAGATCCTGATTTGGT-3'
cry1a-F1	5'-ATTAGTTTCAACCACCCCTACAGT-3'
cry1a-R1	5'-CCTCATCCCAGCATCCACTA-3'
