# Supplementary material for: Hormone Regulation Effect of Blue Light on Soybean Stem Internode Growth Based on the Grey Correlation Analysis Model
Source: Int J Mol Sci. 2025 May 6;26(9):4411. doi: 10.3390/ijms26094411 (PMC12072184; doi:10.3390/ijms26094411)
Supplement: Supplementary file 1 [file ijms-26-04411-s001.zip › Supplementary material S2.pdf]

Proteomic analysis：

Materials and methods

1. Experiments

1.1 Protein extraction and digestion

SDT(4%SDS，100mM Tris-HCl，1mM DTT，pH7.6) buffer was used for sample lysis and protein extraction. The amount of protein was quantified with the BCA Protein Assay Kit (Bio-Rad, USA). Protein digestion by trypsin was performed according to filter-aided sample preparation (FASP) procedure described by Matthias Mann. The digest peptides of each sample were desalted on C18 Cartridges (Empore™ SPE Cartridges C18 (standard density), bed I.D. 7 mm, volume 3 ml, Sigma), concentrated by vacuum centrifugation and reconstituted in 40 µl of 0.1% (v/v) formic acid.

Filter-aided sample preparation (FASP Digestion) procedure：200 µg of proteins for each sample were incorporated into 30 µl SDT buffer (4% SDS, 100 mM DTT, 150 mM Tris-HCl pH 8.0). The detergent, DTT and other low-molecular-weight components were removed using UA buffer (8 M Urea, 150 mM Tris-HCl pH 8.0) by repeated ultrafiltration (Microcon units, 10 kD). Then 100 µl iodoacetamide (100 mM IAA in UA buffer) was added to block reduced cysteine residues and the samples were incubated for 30 min in darkness. The filters were washed with 100 µl UA buffer three times and then 100 µl 25mM NH4HCO3 buffer twice. Finally, the protein suspensions were digested with 4 µg trypsin (Promega) in 40 µl 25mM NH4HCO3 buffer overnight at 37 °C, and the resulting peptides were collected as a filtrate. The peptides of each sample were desalted on C18 Cartridges (Empore™ SPE Cartridges C18 (standard density), bed I.D. 7 mm, volume 3 ml, Sigma), concentrated by vacuum centrifugation and reconstituted in 40 µl of 0.1% (v/v) formic acid. The peptide content was estimated by UV light spectral density at 280 nm using an extinctions coefficient of 1.1 of 0.1% (g/l) solution that was calculated on the basis of the frequency of tryptophan and tyrosine in vertebrate proteins.

1.2 SDS-PAGE

20 µg of protein for each sample were mixed with 5X loading buffer respectively and boiled for 5 min. The proteins were separated on 12.5%

SDS-PAGE gel (constant current 14 mA, 90 min). Protein bands were visualized by Coomassie Blue R-250 staining.

1.3 Labeling

iTRAQ ： 100 μg peptide mixture of each sample was labeled using iTRAQ reagent according to the manufacturer's instructions (Applied Biosystems).

TMT ： 100 μg peptide mixture of each sample was labeled using TMT reagent according to the manufacturer's instructions (Thermo Scientific).

1.4 High pH Reversed-Phase Fractionation

Labeled peptides were fractionated by High pH Reversed-Phase Peptide Fractionation Kit (Thermo Scientific). The dried peptide mixture was reconstituted and acidified with 0.1% TFA solution and loaded to the equilibrated, high-pH, reversed-phase fractionation spin column. Peptides are bound to the hydrophobic resin under aqueous conditions and desalted by washing the column with water by low-speed centrifugation. A step gradient of increasing acetonitrile concentrations in a volatile high-pH elution solution is then applied to the columns to elute bound peptides into 10 different fractions collected by centrifugation. The collected fractions were desalted on C18 Cartridges (Empore™ SPE Cartridges C18 (standard density), bed I.D. 7 mm, volume 3 ml, Sigma) and concentrated by vacuum centrifugation.

1.5 LC-MS/MS analysis

LC-MS/MS analysis was performed on a Q Exactive mass spectrometer (Thermo Scientific) that was coupled to Easy nLC (Proxeon Biosystems, now Thermo Fisher Scientific) for 60/90 min. The peptides were loaded onto a reverse phase trap column (Thermo Scientific Acclaim PepMap100, 100 μm*2 cm, nanoViper C18) connected to the C18-reversed phase analytical column (Thermo Scientific Easy Column, 10 cm long, 75 μm inner diameter, 3μm resin) in buffer A (0.1% Formic acid) and separated with a linear gradient of buffer B (84% acetonitrile and 0.1% Formic acid) at a flow rate of 300 nl/min controlled by IntelliFlow technology. The mass spectrometer was operated in positive ion mode. MS data was acquired using a data-dependent top10 method dynamically choosing the most abundant precursor ions from the survey scan (300–1800 m/z) for HCD fragmentation. Automatic gain control (AGC) target was set to 3e6, and maximum inject time to 10 ms. Dynamic exclusion duration was 40.0 s. Survey

scans were acquired at a resolution of 70,000 at m/z 200 and resolution for HCD spectra was set to 17,500 at m/z 200, and isolation width was 2 m/z. Normalized collision energy was 30 eV and the underfill ratio, which specifies the minimum percentage of the target value likely to be reached at maximum fill time, was defined as 0.1%. The instrument was run with peptide recognition mode enabled.

1.6 Identification and quantitation of proteins

The MS raw data for each sample were searched using the MASCOT engine (Matrix Science, London, UK; version 2.2) embedded into Proteome Discoverer 1.4 software for identification and quantitation analysis.

2. Bioinformatic analysis

2.1 Cluster analysis

Cluster 3.0 (http://bonsai.hgc.jp/~mdehoon/software/cluster/software.htm) and Java Treeview software (http://jtreeview.sourceforge.net) were used to performing hierarchical clustering analysis. Euclidean distance algorithm for similarity measure and average linkage clustering algorithm (clustering uses the centroids of the observations) for clustering were selected when performing hierarchical clustering. A heat map was often presented as a visual aid in addition to the dendrogram.

2.2 Subcellular localization

CELLO (http://cello.life.nctu.edu.tw/) which is a multi-class SVM classification system, was used to predict protein subcellular localization.

2.3 Domain annotation

Protein sequences are searched using the InterProScan software to identify protein domain signatures from the InterPro member database Pfam.

2.4 GO annotation

The protein sequences of the selected differentially expressed proteins were locally searched using the NCBI BLAST+ client software (ncbi-blast-2.2.28+-win32.exe) and InterProScan to find homologue sequences, then gene ontology (GO) terms were mapped and sequences were annotated using the software program Blast2GO. The GO annotation results were plotted by R scripts.

2.5 KEGG annotation

Following annotation steps, the studied proteins were blasted against the online Kyoto Encyclopedia of Genes and Genomes (KEGG) database

(http://geneontology.org/) to retrieve their KEGG orthology identifications and were subsequently mapped to pathways in KEGG.

2.6 Enrichment analysis

Enrichment analysis were applied based on the Fisher' exact test, considering the whole quantified proteins as background dataset. Benjamini-Hochberg correction for multiple testing was further applied to adjust derived p-values. And only functional categories and pathways with p-values under a threshold of 0.05 were considered as significant.

2.7 Protein-protein interaction analysis

The protein–protein interaction (PPI) information of the studied proteins was retrieved from IntAct molecular interaction database [11] (http://www.ebi.ac.uk/intact/) by their gene symbols or STRING software (http://string-db.org/). The results were downloaded in the XGMML format and imported into Cytoscape software (http://www.cytoscape.org/, version 3.2.1) to visualize and further analyze functional protein-protein interaction networks. Furthermore, the degree of each protein was calculated to evaluate the importance of the protein in the PPI network.
