# Supplementary figures and images for: Hormone Regulation Effect of Blue Light on Soybean Stem Internode Growth Based on the Grey Correlation Analysis Model
Source: Int J Mol Sci. 2025 May 6;26(9):4411. doi: 10.3390/ijms26094411 (PMC12072184; doi:10.3390/ijms26094411)

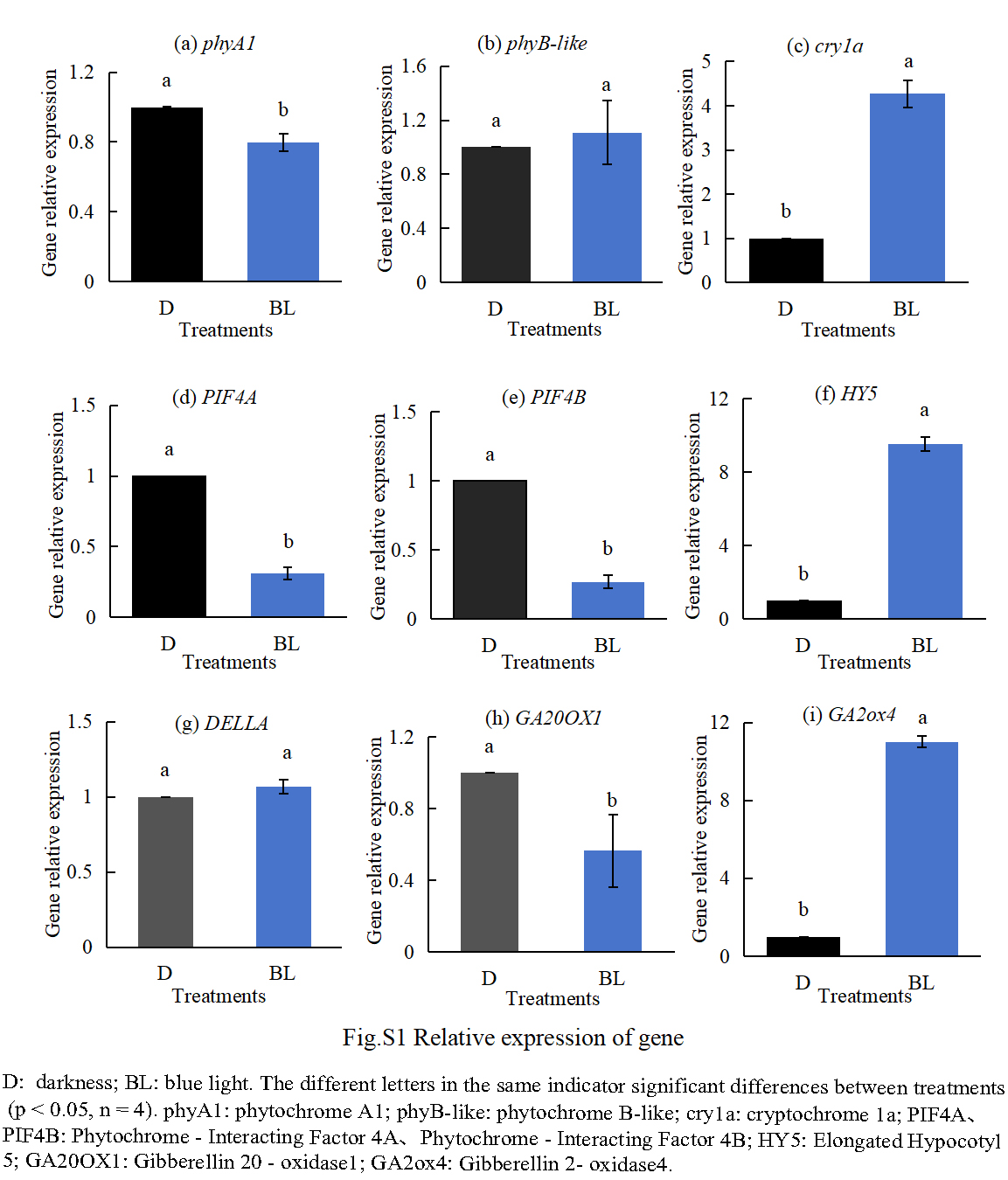

Supplement: Supplementary file 1 [file ijms-26-04411-s001.zip › Fig.S1.jpg]
